# Predictors of non-transport by emergency medical services after a nonfatal opioid overdose: a national analysis

**DOI:** 10.1093/haschl/qxaf101

**Published:** 2025-05-20

**Authors:** Jirka Taylor, Sachini Bandara, Cindy Parks Thomas, Brendan Saloner, Peter James Fredericks, Karen Shen

**Affiliations:** Department of Health Policy and Management, Bloomberg School of Public Health, Johns Hopkins University, 615 N Wolfe St, Baltimore, MD 21205, United States; Department of Mental Health, Bloomberg School of Public Health, Johns Hopkins University, Baltimore, MD 21205, United States; Heller Graduate School for Social Policy and Management, Brandeis University, Waltham, MA 02453, United States; Department of Health Policy and Management, Bloomberg School of Public Health, Johns Hopkins University, 615 N Wolfe St, Baltimore, MD 21205, United States; Department of Emergency Medicine, School of Medicine, Johns Hopkins University, Baltimore, MD 21205, United States; Department of Health Policy and Management, Bloomberg School of Public Health, Johns Hopkins University, 615 N Wolfe St, Baltimore, MD 21205, United States

**Keywords:** emergency response, opioid overdose, nonfatal overdose, linkage to care

## Abstract

**Introduction:**

Emergency medical services (EMS) response to nonfatal overdoses represents an opportunity to provide linkage to services. However, patients may decline follow-on transportation. This paper examined predictors associated with non-transport after nonfatal opioid overdoses.

**Methods:**

We undertook a cross-sectional analysis of 2021-2022 data from the National EMS Information System. The outcome was a binary measure of declined transport. Predictors included age, gender, race/ethnicity, response time, EMS level of care, naloxone does, setting, urbanicity, region, time, and day.

**Results:**

We identified 710 263 nonfatal opioid overdose events, of which 12.4% ended in non-transport. Men were more likely to decline transport (average marginal effect [AME] 0.020 [95% CI, 0.019-0.022]), and Black patients were less likely to decline (AME −0.022 [95% CI, −0.024 to −0.021]). Compared with events involving an EMS-administered limited first dose (<2 mg), non-transports were more likely in events without naloxone administration (AME 0.075 [95% CI, 0.073-0.078]), with administration only by laypersons (AME 0.101 [95% CI, 0.096-0.107]), and when EMS administered higher first doses (2-4 mg AME 0.039 [95% CI, 0.037-0.041]; 4 mg+ AME 0.053 [95% CI, 0.049-0.056]).

**Conclusion:**

Efforts to improve post-overdose care should focus on groups who are more likely to decline transport and on appropriate naloxone dosing.

## Introduction

Between 2021 and 2023, there were approximately 80 000 annual fatal drug overdoses involving opioids in the United States, but this is far surpassed by the occurrence of nonfatal overdoses.^1,2^ Given elevated risks of subsequent mortality among people who survive an overdose, nonfatal overdoses offer an opportunity to engage individuals at a critical moment and offer treatment and other support services.^3,4^ Emergency medical services (EMS) are often the first point of medical contact for a person experiencing an overdose.^5,6^ EMS may administer naloxone to reverse respiratory depression. Standard of care recommends that patients should subsequently be transported to a hospital emergency department (ED) for follow-on treatment for acute medical needs and referral to longer-term treatment.^7-9^ However, patients who receive EMS care for an opioid overdose may decline transport to the hospital, preventing the continuation of care in the hospital and linkage to follow-on care. Patients may decline transport to the hospital for several reasons. They may believe that they do not require further care, anticipate costly or poor quality in-hospital care, or have negative experiences with first responders.^10-12^ In cases where naloxone is administered, there is a risk that patients experience precipitated withdrawal, wherein naloxone induces immediate withdrawal symptoms such as severe nausea, vomiting, diarrhea, and dysphoria.^13^ This risk is higher if high naloxone doses are used.^14^ The discomfort of precipitated withdrawal may cause patients to decline further treatment.

Research examining predictors of non-transport after nonfatal overdoses is very rare,^15^ and the measurement of non-transport rates is highly inconsistent. The only nationwide estimate dates back to 2016, which found a rate of non-transport of less than 4%.^16^ However, this estimate precedes substantial changes in the illicit opioid supply and availability of services to people who use opioids, including increased availability of naloxone to laypersons. More recent studies offer much higher estimates ranging from 10% to 40%; however, these are generally smaller-scale studies using data from a single jurisdiction or agency.^15,17-21^ A better understanding of the overall rate of non-transport and predictors of non-transport can improve our understanding of the scope of the overdose crisis and better target interventions to improve EMS response.

We analyzed a national database of EMS encounters in 2021-2022 to produce an updated national estimate of the non-transport rate and to identify correlates and trends in non-transports after nonfatal opioid overdoses.

## Methods

### Data source

We analyzed 2021-2022 data from the National EMS Information System (NEMSIS), a national database of EMS activities in the United States organized by the National Highway Traffic Safety Administration.^22^ Data are provided to NEMSIS on a voluntary basis by nearly 14 000 participating EMS agencies,^23^ comprising approximately 95% of all EMS agencies nationwide that “respond to 911 calls for emergency care and transport to acute care facilities”.^24^ NEMSIS is derived from patient care reports filled out by EMS personnel after interacting with a patient and has been used in a variety of existing studies focusing both on opioid-related^25,26^ and non-opioid-related topics.^27-29^

### Study population

Our study sample consisted of events involving EMS response to a nonfatal opioid overdose. We begin by identifying all nonfatal EMS adult encounters by excluding (1) events involving people under age 18, (2) events with dispositions that did not indicate an encounter with a patient or that indicated transfer to another EMS unit, (3) events where the patient died, and (4) a small number of observations where patients were released per protocol, indicating that transport was not deemed necessary. For encounters with multiple responding agencies, we only included data from the final responding agency (see Supplementary Appendix for more details).^24^

Following an approach used in the NEMSIS nonfatal opioid overdose tracker,^24^ we then defined an opioid overdose as an event with at least one of the following characteristics:

1) The primary or secondary provider impression (ie, clinical assessment by EMS personnel of the patient's primary and secondary problem that led to the encounter) contains an ICD-10 code for opioid use disorder or opioid poisoning (F11, T40.1-40.6, T40.9).

OR

2) The event includes an administration of naloxone and a positive response to the administration, which highly suggests opioid poisoning.

More details are provided in the Supplementary Appendix.

### Outcome measure

Our main outcome variable was a binary indicator for non-transport, defined via the “patient disposition” variable in NEMSIS. Our primary outcome variable combines 2 disposition types: people who declined both treatment and transport (36.6% of non-transports), and people who received treatment but then declined transport for follow-up care (63.4% of non-transports). In Table S1, we display analyses considering these events separately.

### Key covariates of interest

We considered the relationship between non-transport and 3 types of covariates. First, we included socio-demographic characteristics reported by EMS (gender, race/ethnicity, age). Second, we included event-based characteristics that may have impacted the likelihood of declining transport such as setting, day-of-the-week, time-of-day, major census region, and county rurality of response location (based on USDA Urban Influence codes). Third, we examined the relationship between patients declining transport and features of the EMS response including response time, level of responding EMS unit (advanced [ALS], denoting the ability to provide more complex care vs basic life support [BLS]), whether naloxone was administered by EMS and how much naloxone was used. For naloxone administration, we group patients into 5 categories: no naloxone administered, naloxone administered by bystanders only, and 3 categories for cases where EMS administered naloxone based on the dose of the first administration: <2, 2-4, and 4 mg or more. We choose to consider the first administration as an indicator of whether the EMS provider used low or high doses because, in cases of multiple administrations, the use of high doses may indicate that the first dose was unsuccessful. Our dosage thresholds are based on the 2 doses of intranasal naloxone that were available for our entire sample period (2 and 4 mg). A new 8 mg formulation was approved in April 2021.

### Analytical approach

We first described characteristics of all nonfatal EMS encounters in the dataset, separately for opioid overdose encounters and non-opioid overdose encounters. Second, we analyzed the trend in the rate of non-transport by summarizing the average rate of non-transport among all opioid overdose encounters by month, and then separately by type of non-transport (with or without care refusal). Third, we summarized the probability of non-transport across the selected covariates of interest, broken down by non-transport types, to obtain unadjusted univariate comparisons of non-transport. Finally, we included all covariates in a multivariate logistic regression that also included calendar month and state fixed effects. In our primary specification, we limited our set of variables to only those with low rates of missingness (less than 10% with the exception of race/ethnicity, missingness rates are shown in Supplementary Appendix), out of concern for possible selective missingness correlated with our outcome variable of declining transport, eg, if EMS responders were less likely to fill these variables out in cases of a non-transport. We used a categorical indicator approach to address missing data in the main model. We also performed a complete case analysis and an analysis using multiple imputation in sensitivity analyses as alternative ways to handle missing data. The work was approved by the Johns Hopkins Bloomberg School of Public Health IRB and is reported in accordance with STROBE guidelines.

## Results

In 2021 and 2022, there were 710 263 nonfatal opioid overdose events reported in NEMSIS (Table 1). Of these events, 88 111 (12.4%) ended with the patient declining transport. These opioid overdose events affected a younger and predominantly male population (39% of patients were less than 36 years old and 68% were male). About a third (32%) of events did not involve any naloxone administration, and an additional 12% had naloxone administered only prior to EMS arrival (eg, by a bystander). Of the remaining, first naloxone doses of at least 2 mg but less than 4 mg were most common, followed by doses of <2 mg. Approximately 5% of our sample received a first naloxone dose of ≥ 4 mg. The non-transport rate for opioid overdose events grew over time, from 10.6% in January 2021 to 14.1% in December 2022 (*P* < .001) (Figure S1).

**Table 1.** Characteristics of opioid overdose encounters.

Predictor	Opioid overdose sample (*n* = 710 263)
Non-transport rate	
Overall	88 111 (12.4%)
*Declined care and transport*	*32 257 (4.5%)*
*Declined transport only*	*55 854 (7.9%)*
Gender	
Female	225 226 (31.8%)
Race/ethnicity	
White	381 186 (63.8%)
Black/African American	144 278 (24.2%)
Hispanic or Latino	54 585 (9.1%)
Other	17 211 (2.9%)
Age	
18-35 years	278 164 (39.4%)
36-55 years	274 557 (38.9%)
56+ years	153 343 (21.7%)
Censored	497 (0.1%)
Region	
Northeast	125 175 (17.6%)
Midwest	180 843 (25.5%)
South	292 360 (41.2%)
West	111 858 (15.7%)
Urbanicity	
Rural	36 715 (5.3%)
Response time (min)	
First quartile (0-4 min)	168 938 (23.8%)
Second quartile (4-6 min)	181 945 (25.7%)
Third quartile (6-8 min)	154 614 (21.8%)
Fourth quartile (8-18 min)	167 941 (23.7%)
Censored	35 399 (5.0%)
First naloxone dose	
No administration	209 982 (32.3%)
Lay administration only	16 792 (2.6%)
Less than 2 mg	135 619 (20.9%)
≥2 mg, <4 mg	235 953 (36.3%)
4 mg or more	48 536 (7.5%)
Censored	2377 (0.4%)
Level of care	
ALS	630 139 (88.7%)
Location	
Private residence	352 515 (51.5%)
Street/road	134 315 (19.6%)
Commercial	70 375 (10.3%)
Other	127 641 (18.6%)
Time of day	
Day (6 Am-6 Pm)	352 123 (49.6%)
Day of week	
Weekend	314 647 (44.3%)
Initial acuity*	
Lower	179 919 (35.4%)
Emergent	215 496 (42.4%)
Critical	113 306 (22.3%)
Final acuity*	
Lower	273 521 (59.0%)
Emergency	159 786 (34.5%)
Critical	30 187 (6.5%)
Responsiveness*	
Only responsive measurements	385 698 (64.0%)
Only unresponsive measurements	156 306 (10.1%)
Both responsive and unresponsive measurements	61 106 (25.9%)
Payment*	
Insurance	69 581 (19.5%)
Medicaid	41 423 (11.6%)
Medicare	18 568 (5.2%)
Other	43 293 (12.1%)
Self-pay	65 402 (18.3%)
No insurance	119 249 (33.4%)

Source: Authors’ analysis of data from the National EMS Information System, 2021-2022.

Sample characteristics are presented for the study population of overdose encounters included in the NEMSIS dataset. Variable definitions are presented in the Supplementary Appendix. Descriptive statistics for each variable are based on the sample for which the variable is nonmissing. Acuity, responsiveness, and payment variables each had substantial missingness, indicated by the asterisk in the table: rates of missingness for each variable are summarized in Table S2. We censored implausible values (see Supplementary Appendix).

Abbreviation: ALS, advanced life support.

**Figure 1.**
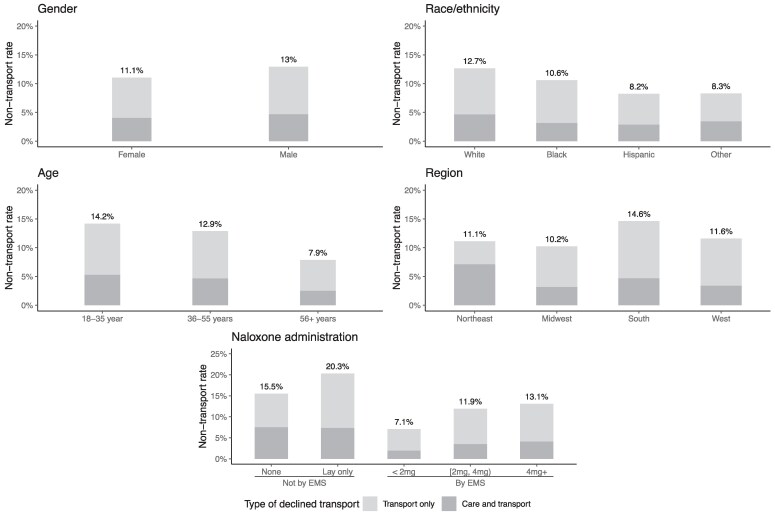
Unadjusted non-transport rate among opioid overdose emergency medical services (EMS) encounters, by key patient-, place-, and event characteristics. Source: Authors’ analysis of data from the National EMS Information System, 2021-2022. Each panel shows the percent of nonfatal opioid overdose events (*N* = 710 263) that ended in a non-transport, splitting the sample by a patient-, place-, or event-based characteristic of interest.

Male, White, and younger patients had the highest rates of non-transport (Figure 1). 13.0% of male patients declined transport, compared with 11.1% of female patients (*P* < .001), and 12.7% of White patients declined transport, compared with 10.6% of Black patients and 8.2% of Hispanic patients (*P* < .001). 14.2% of patients under 35 declined transport, compared with 12.7% for people 36-55, and 7.8% for people over 55 (*P* < .001). Overdoses taking place in the South had a significantly higher non-transport rate than those in other regions (14.6% in the South, 11.1% in the Northeast, 10.2% in the Midwest, and 11.6% in the West) (*P* < .001). With respect to naloxone administration, we found the highest rates of declined transport in cases where naloxone was not administered (15.5%), or was administered only by bystanders (20.3%). Among events where EMS administered naloxone, higher first doses were associated with greater rates of declined transport. Transport was declined in 7.1% of cases where the first dose was under 2 mg, 11.9% of cases with a first dose of 2-4 mg, and 13.1% of cases where the first dose was 4 mg or higher (*P* < .001).

Table 2 shows that the above relationships remain very similar after adjustment for other covariates in the full model. After full covariate adjustment, there was also a significantly higher likelihood of non-transport for overdoses occurring in private residences (compared with other locations) and for events with shorter EMS response times. The probability of a non-transport was also slightly higher for events occurring in nonrural locations, those attended by ALS units, and events taking place at night. There were no differences between events occurring on a weekday and on a weekend.

**Table 2.** Predictors of non-transport among nonfatal opioid overdose, unadjusted non-transport rates, and average marginal effects from adjusted analysis.

Predictor	Unadjusted non-transport rate (%)	Average marginal effect [95% CI]
Gender		
Female	11.1	Ref
Male	13.0	0.020 [0.019-0.022]
Race/ethnicity		
White	12.7	Ref
Black/African American	10.6	−0.022 [−0.024 to −0.020]
Hispanic or Latino	8.2	−0.025 [−0.028 to −0.022]
Other	8.3	−0.029 [−0.034 to −0.024]
Age		
18-35 years	13.9	Ref
36-55 years	12.7	−0.008 [−0.010 to −0.006]
56+ years	7.8	−0.050 [−0.051 to −0.048]
Urbanicity		
Rural	12.6	Ref
Nonrural	12.4	0.019 [0.011-0.027]
Response time		
First quartile (0-4 min)	14.1	Ref
Second quartile (4-6 min)	12.4	−0.017 [−0.019 to −0.014]
Third quartile (6-8 min)	11.6	−0.029 [−0.032 to −0.027]
Fourth quartile (8-18 min)	11.1	−0.043 [−0.046 to −0.041]
First naloxone dose		
No administration	15.5	Ref
Lay administration only	20.3	0.026 [0.020, 0.031]
Less than 2 mg	7.1	−0.076 [−0.078 to −0.073]
≥2 mg, <4 mg	11.9	−0.036 [−0.038 to −0.034]
4 mg or more	13.1	−0.023 [−0.026 to −0.020]
Level of care		
BLS	15.1	Ref
ALS	12.1	0.003 [0.001-0.006]
Location		
Private residence	13.2	Ref
Street/road	12.1	−0.027 [−0.029 to −0.025]
Commercial	14.3	−0.008 [−0.010 to −0.005]
Other	9.4	−0.051 [−0.053 to −0.049]
Time of day		
Day	11.9	Ref
Night	12.8	0.003 [0.001-0.004]
Day of week		
Weekday	12.4	Ref
Weekend	12.3	−0.001 [−0.003 to 0.000]

Source: Authors’ analysis of data from the National EMS Information System, 2021-2022.

Missing data indicators and month-year and state fixed effects are omitted from the table. Average marginal effects come from a logistic regression with other covariates included in the model.

Abbreviations: ALS, advanced life support; BLS, basic life support.

**Figure 2.**
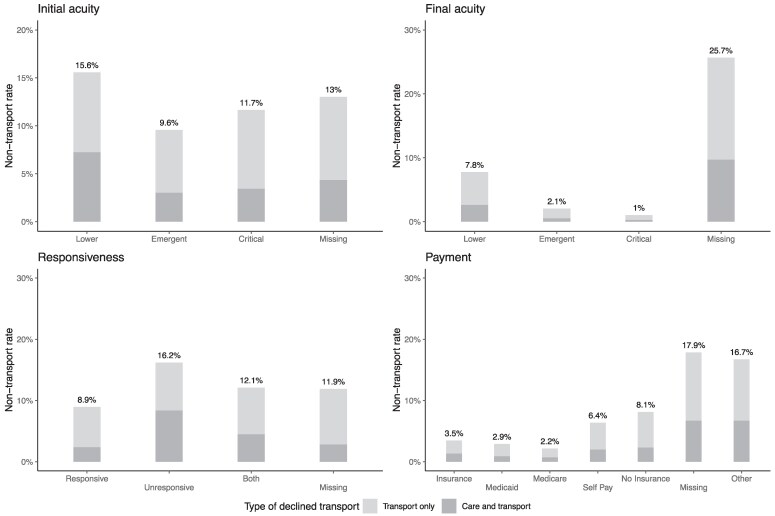
Unadjusted non-transport rate among opioid overdose emergency medical services (EMS) encounters, by other variables of interest. Source: Authors’ analysis of data from the National EMS Information System, 2021-2022. Each panel shows the percent of nonfatal opioid overdose events (*N* = 710 263) that ended in a non-transport, splitting the sample by a variable of interest. 28.4% observations missing for initial acuity, 34.7% for final acuity, 15.1% for responsiveness, and 49.7% for payment. See Supplementary Appendix for additional details on these variables.

As discussed above, our primary specification was limited to covariates with little missingness. However, we hypothesize that patient acuity (categorization of the severity of the patient's condition and their needs), responsiveness (a measure of consciousness), and insurance status may be important predictors of declining transport. In Figure 2, we summarize rates of declining transport by these variables, including the missing category. In general, we found high rates of non-transport among events where these variables are missing, consistent with our concern of selective missingness. However, among people where acuity was reported, we found that patients who are rated by the EMS provider as low acuity were the most likely to decline transport, for both initial and final acuity. Among people where responsiveness was reported, we found patients whose measurements only noted them as unresponsive at any point during the encounter were less likely to decline transport. Among patients where insurance status was reported in the dataset (approximately 50% of observations), we found that non-transport rates were more than twice as high among those with no insurance compared to those having either private insurance, Medicare, or Medicaid. Including these variables in our model of non-transports does not significantly alter our primary findings (Table S1).

### Sensitivity analyses

Our results were robust to analyzing different types of non-transport separately (Table S2), examining events identified via naloxone administration vs provider impression separately (Table S3), and when stratifying by initial patient acuity (Table S4). Results from a complete case analysis (Table S5) and from analyses using an imputed dataset (Tables S6 and S7) also show similar findings.

## Discussion

In an analysis of overdose events attended to by EMS in 2021-2022, we found that patients declined transport to the hospital in 12.4% of cases, that this rate increased over our study period, and that non-transports were more common among male, White, younger patients, patients living in the South, and in cases where EMS did not administer naloxone or administered higher first doses. The 12.4% non-transport rate found in this study is at the lower end of estimates from smaller samples^17-21^, but notably higher than the results from a previous analysis of the 2016 NEMSIS, which found a non-transport rate of less than 4%.^16^ The difference from the prior national study may reflect both a change in the frequency of declined transports over time, as well as methodological differences, primarily the fact that the earlier study focused exclusively on events involving naloxone administrations and had to rely on EMS data with less complete geographical coverage. The earlier study also precedes notable changes in the environment in which opioid overdoses take place, such as the emergence of illegally manufactured synthetic opioids and much increased availability of naloxone in the community.

The substantial and increasing rate of non-transport is concerning as it means a significant portion of people experiencing nonfatal overdose are not receiving key interventions that can be provided in emergency department settings. This includes programs to initiate medications for opioid use disorder (MOUD), provide peer support, or make referrals to community-based services for nonfatal overdose survivors in the ED,^30-35^ which have been associated with improved treatment outcomes and lower representation in the ED.^36-40^ While a number of studies found no evidence of an increased risk of life-threatening events shortly after people declined transport by emergency responders,^20,21,41-43^ multiple studies have found that non-transports were associated with an increased risk of another drug overdose over subsequent months.^18,44^

Our findings suggest multiple potential avenues policymakers could pursue in order to reduce non-transports, and also highlight the populations most likely to benefit from such interventions. For example, our finding of higher rates of declining transport among people treated with high first doses of naloxone is consistent with evidence that titrating naloxone doses can help avoid precipitated withdrawal,^45-47^ and may point to a need for improved guidelines and training for EMS care for people who use drugs.^48,49^ The fact that we also found higher rates of non-transport when naloxone is administered by bystanders may also suggest the need to educate patients on the need for transport for follow-up care after naloxone administration. This education could be folded into existing communication campaigns encouraging naloxone use.^50^ Finally, our finding that rates of non-transport are highest among uninsured patients (where insurance is reported) is consistent with the broader literature on care avoidance in suggesting cost concerns as a key factor in explaining declining further care.^51-53^ We also found substantially higher rates of non-transports in the South, where the majority of states that have not expanded Medicaid are concentrated. Together, these results may suggest that improving health insurance coverage could potentially reduce non-transports.

Our findings may also suggest a need to either improve ED care or to offer alternatives to ED-based care. Patients may be declining transport because they anticipate negative provider attitudes in the ED and poor care.^54^ Indeed, many nonfatal overdose survivors fail to receive evidence-based care in the ED.^39,55-57^ Interventions such as pre-hospital initiation of MOUD by EMS professionals^4,58^ or utilization of crisis stabilization centers with their supportive and compassionate environment^59,60^ could mitigate or address the potential negative impact of declined transports. Further developing the evidence base surrounding alternative response models and any challenges or implications for EMS protocols represents an important area of future research.

Importantly, our findings highlight the potential limitations of relying on hospital claims data as a measure of nonfatal overdose. Given that about 1 in 8 people in our study declined hospital transport after experiencing an opioid overdose, efforts to study the overdose crisis using hospital claims data will underestimate the prevalence of overdose. Our analysis highlights that the scope of this missed reporting varies by geography and patient characteristics. This is an opportunity for local and state governments to improve surveillance efforts by including this data in their monitoring, and explore alternative data collection strategies to capture the experiences of populations who decline transport.

In summary, our analysis shows that a notable share of EMS responses to nonfatal opioid overdoses do not result in transportation to a hospital, with the potentially negative consequences of foregoing hospital-based care and subsequent linkage to other services. Efforts to improve post-overdose care should focus on populations most likely to decline transport by addressing patients’ reasons for not wanting further engagement with healthcare professionals. Avenues that may merit attention include improving EMS care, particularly during naloxone administrations, and offering alternatives to the ED as a transport destination.

### Limitations

These findings are subject to limitations. NEMSIS does not contain a patient identifier, and we thus cannot measure whether patients have previously interacted with EMS, which may be important in explaining declined transports. The lack of a patient identifier also means that the same encounter could appear multiple times in the dataset if multiple agencies responded to the same event. We use a deduplication approach consistent with NEMSIS guidance to minimize this issue, but we may not have removed all duplicated observations. The dataset also lacks information on the quality of care, including patient experience during the EMS encounter or during prior interactions with healthcare professionals, which may be an important factor for people's willingness to engage in post-overdose care. NEMSIS also does not include patient care report narratives, which may include important information about the event that is not captured elsewhere. Lastly, an important caveat to our results is that we examine rates of declining transport conditional on calling 911, and there may be notable differences across patient groups in their inclination to call 911 in the event of an overdose.

## Supplementary Material

qxaf101_Supplementary_Data
