# Supplementary material for: Predictors of non-transport by emergency medical services after a nonfatal opioid overdose: a national analysis
Source: Health Aff Sch. 2025 May 20;3(5):qxaf101. doi: 10.1093/haschl/qxaf101 (PMC12123062; doi:10.1093/haschl/qxaf101)
Supplement: qxaf101_Supplementary_Data [file qxaf101_supplementary_data.zip › Non-transport_predictors_online_supplement_revised_clean.docx]

### Online-only supplements

eAppendix 1: Additional methodological details

eFigure 1

eTable 1

eTable 2

eTable 3

eTable 4

eTable 5

eTable 6

eTable 7

### **eAppendix 1. Additional methodological details**

#### **Deduplication**

NEMSIS is a database of EMS activations, not EMS patients or 911 calls. In other words, each unique ID in the dataset represents an agency responding to a 911 call. Thus, if more than one EMS agency responds to the same 911 call and both agencies report to NEMSIS, there will be multiple events in the dataset associated with this call. Because the data do not contain patient identifiers, there is no way of identifying these duplicate events. In order to deduplicate the dataset to the extent possible, we limited the data to events with dispositions indicating that the agency made contact with a living patient by removing activations such as those where an EMS unit was on standby or was assisting another unit and where the initial acuity or disposition variable indicated that the patient died.

Further, in line with NEMSIS TAC’s deduplication approach, we removed observations with a disposition “transferred to another EMS unit” where the responding unit was recorded as the first on the scene or the value for this variable was not recorded. The objective behind this approach is to retain only one observation per 911 call by utilizing the abovementioned combination of values to identify observations that are likely succeeded in the dataset by another observation pertaining to the same 911 call. One practical implication of this approach is that it results in the retention of the record corresponding to the last EMS unit interacting with the patient. This is noteworthy because one of our predictors of interest is the level of care of the responding unit and this information is likely more important with respect to who treats the patient as opposed to who transports the patient. Since in some cases these are likely two different units, our deduplication approach, which maximizes the chance of retaining the transporting unit for each event, means losing some value from including the level of care variable in our analyses.

#### **Identifying opioid overdose events**

Note that, in contrast with some other studies focusing exclusively on naloxone administration,^1,2^ our main working definition of an opioid overdose does not require naloxone to be administered. Instead, a relevant provider impression is sufficient. This is consistent with NEMSIS’s approach ^3^ and also with literature suggesting that using naloxone only was a relatively poor indicator of opioid overdoses.^4,5^ The broader overdose definition allows for the fact that some overdose response does not require the administration of naloxone, and for cases in which naloxone administration was not recorded. Below, we summarize the percent of our sample that was identified via the impression criterion only, naloxone criterion only, and both criteria.

| **Proportion of events by definition** |  |
| --- | --- |
| % meeting both criteria | 28.9% |
| % meeting ICD criterion only | 33.9% |
| % meeting naloxone criterion only | 37.2% |

Our overdose definition departs from NEMSIS’s approach in one minor aspect. NEMSIS also includes events where the protocol used (eProtocol.01) indicates an opioid overdose. As expected, there is strong overlap between the protocol variable and provider impressions or naloxone administrations. There were only 3,790 events with the opioid overdose protocol but not meeting our main definition. We decided against including these events for reasons of parsimony owing to the overall small number of events involved and also due to the fact that the eProtocol.01 variable also includes a general “overdose” value, which is indicated much more often than the opioid-specific one but is not part of NEMSIS’s definition. Thus, including one but not the other protocol value might introduce some bias due to reporting inconsistencies across EMS units.

The figure below shows the sample selection procedure.


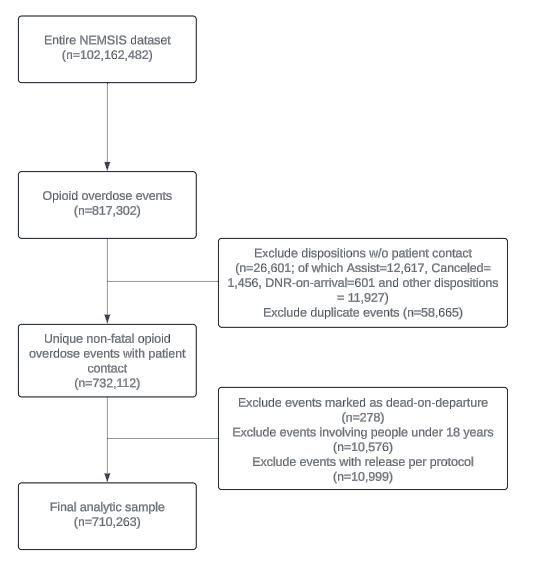


Note: The flowchart shows the number of observations at each stage of sample selection, from all EMS encounters in the entire NEMSIS dataset, to a final analytic sample of unique adult nonfatal opioid overdose encounters where the EMS agency made contact with the patient.

#### **Outcome definition**

The main outcome measure for our analysis was a binary indicator for non-transports using the disposition variable (either receiving care in the field but subsequently declining transport, or declining both care and transport). The dataset also includes a very small number of observations where the patient declined care but was subsequently transported (n=423). We included these event in the overall study population but did not consider them to meet our binary outcome measure. In other words, these events are in the denominator for the overall non-transport rate, but not in the numerator.

#### **Overview of key covariates**

Below, we provide an overview of key covariates of interest, how they appear in the NEMSIS dataset, and how, if at all, they were modified for the purposes of this paper.

| **Covariate** | **Appearance in NEMSIS** | **Use in this paper** | **% Missing or Censored** |
| --- | --- | --- | --- |
| Patient-based characteristics | | |  |
| Age | Continuous (in years) | Implausible values (>99) censored  Categorical (18-35, 36-55, 56+) | 0.6% |
| Gender | Binary (female/male) | Binary (female/male) | 3.7% |
| Race/ethnicity | Categorical (5 categories, multiple categories allowed) | Categorical (single category only, observations with multiple rows coded as “Multiple”) | 15.9% |
| Event-based characteristics | | |  |
| Response time | Continuous (in minutes) | Implausible values (above 95^th^ percentile) censored  Categorical (by quartiles) | 5.2% |
| Naloxone administration | Medications administered categorical  Administration prior to unit arrival binary (yes/no)  Dose (continuous)  Dose units (categorical) | Categorical (5 categories: no administration, only lay administration, less than 2mg, at least 2mg but less than 4mg, 4mg or more); where information on timing of administration was missing, administration assumed to be performed by EMS; dosage of multiple administrations with the same timestamp were averaged; doses higher than 12mg censored | 8.6% |
| Level of EMS unit | Categorical (12 categories) | Binary (advanced life support [ALS]/ basic life support [BLS]) | 0% |
| Place-based characteristics | | |  |
| Census region | Categorical (4 categories) | Categorical (4 categories) | 0% |
| Urbanicity | Categorical (4 categories based on 2013 USDA Urban Influence codes) | Categorical (4 categories) | 2.4% |
| Location type | Categorical (ICD-10 Y92 codes) | Categorical (4 umbrella categories, classification based on NEMSIS guidance ^6^) | 3.7% |
| Variables with high missingness | | | |
| Initial acuity | Categorical (lower, emergent, critical) | Categorical (lower, emergent, critical) | 28.4% |
| Final acuity | Categorical (lower, emergent, critical) | Categorical (lower, emergent, critical) | 34.7% |
| Responsiveness | Categorical (alert, verbal, painful, unresponsive) | Categorical (only responsive (i.e., alert, verbal or painful) measurement(s), only unresponsive measurement(s), both responsive and unresponsive measurement(s)) | 15.1% |
| Payment | Categorical (12 categories) | Categorical (Insurance, Medicaid, Medicare, Other, Self Pay, No Insurance) | 49.7% |

### **References**

1. Geiger C, Smart R, Stein BD. Who receives naloxone from emergency medical services? Characteristics of calls and recent trends. *Substance abuse*. 2020;41(3):400-407.

2. Glenn MJ, Rice AD, Primeau K, et al. Refusals after prehospital administration of naloxone during the COVID-19 pandemic. *Prehospital Emergency Care*. 2020;25(1):46-54.

3. Hoffman C. Nonfatal Drug Overdose Surveillance Dashboard Companion Guide. University of Utah. Accessed March 29, 2024, https://wiki.utahdcc.org/confluence/display/NTD/Nonfatal+Drug+Overdose+Surveillance+Dashboard+Companion+Guide

4. Ajumobi O, Verdugo SR, Labus B, et al. Identification of non-fatal opioid overdose cases using 9-1-1 computer assisted dispatch and prehospital patient clinical record variables. *Prehospital emergency care*. 2022;26(6):818-828.

5. Grover JM, Alabdrabalnabi T, Patel MD, et al. Measuring a crisis: questioning the use of naloxone administrations as a marker for opioid overdoses in a large US EMS system. *Prehospital Emergency Care*. 2018;22(3):281-289.

6. NEMSIS. Suggested and Defined Lists. Accessed March 29, 2024, https://nemsis.org/technical-resources/version-3/version-3-resources/

**eFigure 1. Trend in non-transport rates over time (2021-2022)**


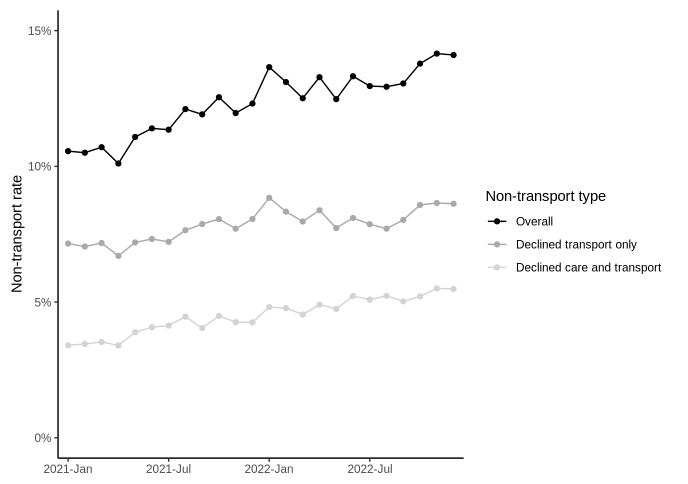


Note: Figure shows the share of nonfatal opioid overdose events in the study population that ended in a non-transport for each month between January 2021-December 2022.

**eTable 1. Comparison of main model results with an extended main model with additional variables with high missingness (initial acuity, final acuity, responsiveness, payment status)**

| **Predictor** | **Main model** | **Extended model** |
| --- | --- | --- |
| **Gender** |  |  |
| Female | Ref | Ref |
| Male | 0.020 [0.019, 0.022] | 0.015 [0.013, 0.016] |
| **Race/ethnicity** |  |  |
| White | Ref | Ref |
| Black/African American | -0.022 [-0.024, -0.020] | -0.013 [-0.015, -0.011] |
| Hispanic or Latino | -0.025 [-0.028, -0.022] | -0.017 [-0.020, -0.014] |
| Other | -0.029 [-0.034, -0.024] | -0.019 [-0.024, -0.014] |
| **Age** |  |  |
| 18-35 years | Ref | Ref |
| 36-55 years | -0.008 [-0.010, -0.006] | -0.005 [-0.006, -0.003] |
| 56+ years | -0.050 [-0.051, -0.048] | -0.035 [-0.037, -0.033] |
| **Urbanicity** |  |  |
| Rural | Ref | Ref |
| Non-rural | 0.019 [0.011, 0.027] | 0.004 [0.001, 0.007] |
| **Response time** |  |  |
| 1^st^ quartile | Ref | Ref |
| 2^nd^ quartile | -0.017 [-0.019, -0.014] | -0.011 [-0.013, -0.009] |
| 3^rd^ quartile | -0.029 [-0.032, -0.027] | -0.018 [-0.020, -0.016] |
| 4^th^ quartile | -0.043 [-0.046, -0.041] | -0.027 [-0.029, -0.025] |
| **First naloxone dose** |  |  |
| No administration | Ref | Ref |
| Lay administration only | 0.026 [0.020, 0.031] | 0.035 [0.030, 0.039] |
| Less than 2mg | -0.076 [-0.078, -0.073] | -0.056 [-0.058, -0.054] |
| >= 2mg, <4mg | -0.036 [-0.038, -0.034] | -0.029 [-0.031, -0.027] |
| 4mg or more | -0.023 [-0.026, -0.020] | -0.018 [-0.021, -0.015] |
| **Level of care** |  |  |
| BLS | Ref | Ref |
| ALS | 0.003 [0.001, 0.006] | 0.007 [0.005. 0.009] |
| **Location** |  |  |
| Private residence | Ref | Ref |
| Street/road | -0.027 [-0.029, -0.025] | -0.021 [-0.022, -0.019] |
| Commercial | -0.008 [-0.010, -0.005] | -0.007 [-0.009, -0.005] |
| Other | -0.051 [-0.053, -0.049] | -0.038 [-0.040, -0.036] |
| **Time of day** |  |  |
| Day | Ref | Ref |
| Night | 0.003 [0.001, 0.004] | 0.001 [0.000, 0.002] |
| **Day of week** |  |  |
| Weekday | Ref | Ref |
| Weekend | -0.001 [-0.003, 0.000] | 0.000 [-0.002, 0.001] |
| **Initial acuity** | **N/A** |  |
| Lower |  | Ref |
| Emergent |  | -0.014 [-0.016, -0.012] |
| Critical |  | 0.011 [0.008, 0.013] |
| **Final acuity** | **N/A** |  |
| Lower |  | Ref |
| Emergent |  | -0.045 [-0.046, -0.044] |
| Critical |  | -0.055 [-0.057, -0.054] |
| **Responsiveness** | **N/A** |  |
| Never unresponsive measurements |  | Ref |
| Both responsive and unresponsive measurements |  | 0.000 [-0.001, 0.002] |
| Only unresponsive measurements |  | -0.008 [-0.010, -0.005] |
| **Payment** | **N/A** |  |
| Insurance |  | Ref |
| Medicaid |  | -0.009 [-0.011, -0.006] |
| Medicare |  | -0.008 [-0.012, -0.004] |
| Other |  | 0.083 [0.079, 0.086] |
| Self Pay |  | 0.032 [0.029, 0.034] |
| No insurance |  | 0.046 [0.044, 0.048] |

Note: Average marginal effects and associated confidence intervals obtained from a logistic regression using the main model with the addition of the four variables with high missingness (n=710,263). Missing data indicators and state and month-year fixed effects omitted from table.

**eTable 2. Association of patient, place, and event characteristics with different types of non-transports**

| **Predictor** | **Declined care and transport** | **Declined transport only** |
| --- | --- | --- |
| **Gender** |  |  |
| Female | Ref | Ref |
| Male | 0.007 [0.006, 0.008] | 0.014 [0.013, 0.016] |
| **Race/ethnicity** |  |  |
| White | Ref | Ref |
| Black/African American | -0.011 [-0.012, -0.010] | -0.013 [-0.015, -0.012] |
| Hispanic or Latino | -0.011 [-0.013, -0.010] | -0.015 [-0.018, -0.013] |
| Other | -0.009 [-0.012, -0.007] | -0.022 [-0.026, -0.018] |
| **Age** |  |  |
| 18-35 years | Ref | Ref |
| 36-55 years | -0.004 [-0.005, -0.003] | -0.005 [-0.007, -0.004] |
| 56+ years | -0.020 [ -0.021, -0.019] | -0.033 [-0.034, -0.031] |
| **Urbanicity** |  |  |
| Rural | Ref | Ref |
| Non-rural | -0.005 [-0.007, -0.003] | 0.017 [0.014, 0.019] |
| **Response time** |  |  |
| 1^st^ quartile | Ref | Ref |
| 2^nd^ quartile | -0.007 [-0.008, -0.005] | -0.011 [-0.013, -0.009] |
| 3^rd^ quartile | -0.010 [-0.012, -0.009] | -0.021 [-0.023, -0.019] |
| 4^th^ quartile | -0.015 [-0.016, -0.014] | -0.031 [-0.033, -0.029] |
| **First naloxone dose** |  |  |
| No administration | Ref | Ref |
| Lay administration only | -0.003 [-0.007, 0.000] | 0.033 [0.028, 0.038] |
| Less than 2mg | -0.049 [-0.051, -0.048] | -0.031 [-0.033, -0.029] |
| >= 2mg, <4mg | -0.035 [-0.036, -0.033] | -0.005 [-0.006, -0.003] |
| 4mg or more | -0.030 [-0.032, -0.028] | 0.005 [0.002, 0.008] |
| **Level of care** |  |  |
| BLS | Ref | Ref |
| ALS | 0.000 [-0.001, 0.002] | 0.004 [0.002, 0.006] |
| **Location** |  |  |
| Private residence | Ref | Ref |
| Street/road | -0.001 [-0.009, -0.006] | -0.022 [-0.024, -0.020] |
| Commercial | -0.007 [-0.009, -0.006] | -0.002 [-0.004, 0.000] |
| Other | -0.170 [-0.019, -0.016] | -0.038 [-0.040, -0.036] |
| **Time of day** |  |  |
| Day | Ref | Ref |
| Night | -0.001 [-0.002, 0.000] | 0.004 [0.002, 0.005] |
| **Day of week** |  |  |
| Weekday | Ref | Ref |
| Weekend | 0.000 [-0.001, 0.002] | -0.001 [-0.002, 0.001] |

Note: Average marginal effects and associated confidence intervals obtained from a logistic regression using a given type of non-transport as the outcome variable and including all presented covariates. Declined care analysis: n=710,263, non-transport rate=4.6%; declined transport only analysis: n=678,006, non-transport rate=8.2%. Missing data indicators and state and month-year fixed effects omitted from table.

**eTable 3. Predictors of non-transports in events meeting the naloxone definition and in events meeting the impression definition**

| **Predictor** | **Naloxone events** | **Impression events** |
| --- | --- | --- |
| **Gender** |  |  |
| Female | Ref | Ref |
| Male | 0.020 [0.019, 0.022] | 0.019 [0.017, 0.021] |
| **Race/ethnicity** |  |  |
| White | Ref | Ref |
| Black/African American | -0.008 [-0.011, -0.006] | -0.031 [-0.033, -0.028] |
| Hispanic or Latino | -0.015 [-0.019, -0.012] | -0.028 [-0.032, -0.024] |
| Other | -0.028 [-0.033, -0.022] | -0.030 [-0.037, -0.023] |
| **Age** |  |  |
| 18-35 years | Ref | Ref |
| 36-55 years | -0.007 [-0.009, -0.005] | -0.008 [-0.011, -0.006] |
| 56+ years | -0.049 [-0.051, -0.046] | -0.043 [-0.046, -0.040] |
| **Urbanicity** |  |  |
| Rural | Ref | Ref |
| Non-rural | 0.025 [0.022, 0.029] | -0.001 [-0.006, 0.004] |
| **Response time** |  |  |
| 1^st^ quartile | Ref | Ref |
| 2^nd^ quartile | -0.013 [-0.015, -0.010] | -0.020 [-0.023, -0.017] |
| 3^rd^ quartile | -0.025 [-0.027, -0.022] | -0.032 [-0.035, -0.029] |
| 4^th^ quartile | -0.036 [-0.039, -0.034] | -0.045 [-0.048, -0.042] |
| **First naloxone dose** |  |  |
| No administration | NA | -0.016 [-0.023, -0.010] |
| Lay administration only | Ref | Ref |
| Less than 2mg | -0.061 [-0.068, -0.055] | -0.081 [-0.087, -0.074] |
| >= 2mg, <4mg | -0.073 [-0.079, -0.067] | -0.037 [-0.044, -0.030] |
| 4mg or more | -0.062 [-0.069, -0.056] | -0.029 [-0.036, -0.021] |
| **Level of care** |  |  |
| BLS | Ref | Ref |
| ALS | -0.020 [-0.023, -0.016] | 0.008 [0.004, 0.011] |
| **Location** |  |  |
| Private residence | Ref | Ref |
| Street/road | -0.013 [-0.015, -0.010] | -0.040 [-0.043, 0.038] |
| Commercial | 0.000 [-0.003, 0.004] | -0.015 [-0.018, -0.011] |
| Other | -0.034 [-0.036, -0.032] | -0.063 [-0.066, -0.060] |
| **Time of day** |  |  |
| Day | Ref | Ref |
| Night | 0.003 [0.001, 0.005] | 0.001 [-0.001, 0.003] |
| **Day of week** |  |  |
| Weekday | Ref | Ref |
| Weekend | -0.002 [-0.003, 0.000] | 0.000 [-0.002, 0.002] |

Note: Average marginal effects and associated confidence intervals obtained from a logistic regression using non-transports as the outcome variable and including all presented covariates, with events matching a given definition as the analytic sample. Naloxone events analysis: n=469,442, non-transport rate=11.1%; impression events analysis: n=446,036, non-transport rate=14.4%. Missing data indicators and state and month-year fixed effects omitted from table.

**eTable 4. Main model stratified by initial acuity category**

| **Predictor** | **Lower** | **Emergent** | **Critical** | **Missing** |
| --- | --- | --- | --- | --- |
| **Gender** |  |  |  |  |
| Female | Ref | Ref |  |  |
| Male | 0.021  [0.018, 0.024] | 0.019  [0.016, 0.021] | 0.023  [0.019, 0.026] | 0.020  [0.017, 0.023] |
| **Race/ethnicity** |  |  |  |  |
| White | Ref | Ref | Ref | Ref |
| Black/African American | -0.042  [-0.046, -0.037] | -0.011  [-0.014, -0.008] | -0.010  [-0.017, -0.006] | -0.020  [-0.024, -0.016] |
| Hispanic or Latino | -0.034  [-0.041, -0.028] | -0.012  [-0.017, -0.007] | -0.024  [-0.031, -0.016] | -0.018  [-0.024, -0.012] |
| Other | -0.040 [-0.050, -0.029] | -0.013  [-0.021, -0.005] | -0.026  [-0.038, -0.015] | -0.030  [-0.040, -0.021] |
| **Age** |  |  |  |  |
| 18-35 years | Ref | Ref | Ref | Ref |
| 36-55 years | -0.011  [-0.015, -0.008] | -0.006  [-0.009, -0.003] | -0.009  [-0.014, -0.005] | -0.005  [0.008, -0.002] |
| 56+ years | -0.051  [-0.055, -0.047] | -0.044  [-0.047, -0.041] | -0.063  [-0.067, -0.058] | -0.041  [-0.045, -0.037] |
| **Urbanicity** |  |  |  |  |
| Rural | Ref | Ref | Ref | Ref |
| Non-rural | -0.016  [-0.025, -0.008] | 0.013  [0.008, 0.018] | 0.026  [0.019, 0.032] | 0.012  [0.006, 0.018] |
| **Response time** |  |  |  |  |
| 1^st^ quartile | Ref | Ref | Ref | Ref |
| 2^nd^ quartile | -0.026  [-0.031, -0.021] | -0.009  [-0.013, -0.006] | -0.008  [-0.013, -0.003] | -0.020  [-0.024, -0.015] |
| 3^rd^ quartile | -0.039  [-0.044, -0.033] | -0.019  [-0.022, -0.015] | -0.019  [-0.025, -0.014] | -0.037  [-0.041, -0.032] |
| 4^th^ quartile | -0.056  [-0.061, -0.051] | -0.028  [-0.032, -0.025] | -0.034  [-0.040, -0.029] | -0.054  [-0.058, -0.050] |
| **First naloxone dose** |  |  |  |  |
| No administration | Ref | Ref | Ref | Ref |
| Lay administration only | 0.054  [0.042, 0.065] | 0.025  [0.015, 0.034] | 0.038  [0.020, 0.056] | 0.011  [0.003, 0.020] |
| Less than 2mg | -0.090  [-0.094, -0.085] | -0.043  [-0.047, -0.039] | -0.052  [-0.060, -0.045] | -0.088  [-0.092, -0.083] |
| >= 2mg, <4mg | -0.022  [-0.027, -0.018] | -0.011  [-0.015, -0.007] | -0.038  [-0.045, -0.031] | -0.038  [-0.042, -0.035] |
| 4mg or more | -0.027  [-0.035, -0.019] | -0.006  [-0.011, 0.000) | -0.026  [-0.035, -0.017] | -0.007  [-0.013, 0.000] |
| **Level of care** |  |  |  |  |
| BLS | Ref | Ref | Ref | Ref |
| ALS | 0.033  [0.029, 0.038] | -0.013  [-0.018, -0.009] | -0.036  [-0.043, -0.028] | 0.022  [0.018, 0.026] |
| **Location** |  |  |  |  |
| Private residence | Ref | Ref | Ref | Ref |
| Street/road | -0.049  [-0.054, -0.045] | -0.023  [-0.026, -0.019] | -0.014  [-0.019, -0.008] | -0.027  [-0.030, -0.023] |
| Commercial | -0.022  [-0.028, -0.017] | 0.000  [-0.004, 0.005] | 0.005  [-0.001, 0.011] | -0.012  [-0.017, -0.008] |
| Other | -0.074  [-0.078, -0.069] | -0.036  [-0.040, -0.033] | -0.027  [-0.032, -0.021] | -0.059  [-0.063, -0.055] |
| **Time of day** |  |  |  |  |
| Day | Ref | Ref | Ref | Ref |
| Night | -0.001  [-0.004, 0.003] | 0.006  [0.004, 0.009] | 0.006  [0.002, 0.010] | -0.001  [-0.003, 0.002] |
| **Day of week** |  |  |  |  |
| Weekday | Ref | Ref | Ref | Ref |
| Weekend | -0.001  [-0.005, 0.002] | 0.000  [-0.003, 0.002] | -0.002  [-0.006, 0.001] | -0.001  [-0.004, 0.002] |

Note: Average marginal effects and associated confidence intervals obtained from a logistic regression using non-transports as the outcome variable and including all presented covariates, with events matching a given acuity level as the analytic sample. Notes: Lower acuity: n=179,919, non-transport rate=15.6%; emergent acuity: n=215,496, non-transport rate=9.6%; critical acuity: n=113,306, non-transport rate=11.7%; missing data: n=201,542, non-transport rate=13.0%. Missing data indicators and state and month-year fixed effects omitted from table.

**eTable 5: Main model using complete case analysis**

| **Predictor** | **Complete case analysis** |
| --- | --- |
| **Gender** |  |
| Female | Ref |
| Male | 0.020 [0.019, 0.022] |
| **Race/ethnicity** |  |
| White | Ref |
| Black/African American | -0.022 [-0.025, -0.020] |
| Hispanic or Latino | -0.017 [-0.020, -0.013] |
| Other | -0.023 [-0.028, -0.018] |
| **Age** |  |
| 18-35 years | Ref |
| 36-55 years | -0.008 [-0.010, -0.006] |
| 56+ years | -0.048 [-0.050, -0.046] |
| **Urbanicity** |  |
| Rural | Ref |
| Non-rural | 0.009 [0.006, 0.013] |
| **Response time** |  |
| 1^st^ quartile (0-4 mins) | Ref |
| 2^nd^ quartile (4-6 mins) | -0.016 [-0.019, -0.013] |
| 3^rd^ quartile (6-8 mins) | -0.027 [-0.030, -0.025] |
| 4^th^ quartile (8-18 mins) | -0.039 [-0.042, -0.037] |
| **First naloxone dose** |  |
| No administration | Ref |
| Lay administration only | 0.019 [0.014, 0.025] |
| Less than 2mg | -0.075 [-0.077, -0.072] |
| >= 2mg, <4mg | -0.041 [-0.043, -0.039] |
| 4mg or more | -0.035 [-0.039, -0.031] |
| **Level of care** |  |
| BLS | Ref |
| ALS | 0.007 [0.004, 0.010] |
| **Location** |  |
| Private residence | Ref |
| Street/road | -0.032 [-0.035, -0.030] |
| Commercial | -0.008 [-0.011, -0.005] |
| Other | -0.050 [-0.052, -0.048] |
| **Time of day** |  |
| Day | Ref |
| Night | 0.003 [0.002, 0.005] |
| **Day of week** |  |
| Weekday | Ref |
| Weekend | -0.001 [-0.003, 0.001] |

Note: Average marginal effects and associated confidence intervals obtained from a logistic regression using non-transports as the outcome variable and including all presented covariates. Complete case analysis n=508,056, non-transport rate = 11.5%. Month-year and state fixed effects omitted from the table.

**eTable 6. Main model using multiple imputation dataset**

| **Predictor** | **Main model, imputed data** |
| --- | --- |
| **Gender** |  |
| Female | Ref |
| Male | 0.020 [0.019, 0.022] |
| **Race/ethnicity** |  |
| White | Ref |
| Black/African American | -0.017 [-0.019, -0.014] |
| Hispanic or Latino | -0.019 [-0.024, -0.014] |
| Other | -0.032 [-0.036, -0.027] |
| **Age** |  |
| 18-35 years | Ref |
| 36-55 years | -0.009 [-0.010, -0.007] |
| 56+ years | -0.050 [-0.052, -0.049] |
| **Urbanicity** |  |
| Rural | Ref |
| Non-rural | 0.012 [0.009, 0.015] |
| **Response time** |  |
| 1^st^ quartile (0-4 mins) | Ref |
| 2^nd^ quartile (4-6 mins) | -0.017 [-0.019, -0.014] |
| 3^rd^ quartile (6-8 mins) | -0.030 [-0.032, -0.028] |
| 4^th^ quartile (8-18 mins) | -0.044 [-0.047, -0.042] |
| **First naloxone dose** |  |
| No administration | Ref |
| Lay administration only | 0.023 [0.018, 0.029] |
| Less than 2mg | -0.069 [-0.071, -0.066] |
| >= 2mg, <4mg | -0.033 [-0.035, -0.031] |
| 4mg or more | -0.021 [-0.025, -0.018] |
| **Level of care** |  |
| BLS | Ref |
| ALS | -0.001 [-0.003, 0.002] |
| **Location** |  |
| Private residence | Ref |
| Street/road | -0.026 [-0.028, -0.024] |
| Commercial | -0.008 [-0.010, -0.005] |
| Other | -0.050 [-0.052, -0.048] |
| **Time of day** |  |
| Day | Ref |
| Night | 0.003 [0.001, 0.004] |
| **Day of week** |  |
| Weekday | Ref |
| Weekend | -0.001 [-0.003, 0.002] |

Note: Average marginal effects and associated confidence intervals obtained from a logistic regression using non-transports as the outcome variable and including all presented covariates (n=710,263). Results are based on a pooled regression of five datasets imputed using the mice package in R with five iterations. Month-year and state fixed effects omitted from the table.

**eTable 7. Extended model incorporating high-missingness variables using multiple imputation dataset**

| **Predictor** | **Extended model, imputed data** |
| --- | --- |
| **Gender** |  |
| Female | Ref |
| Male | 0.019 [0.017, 0.021] |
| **Race/ethnicity** |  |
| White | Ref |
| Black/African American | -0.016 [-0.019, -0.014] |
| Hispanic or Latino | -0.019 [-0.024, -0.015] |
| Other | -0.027 [-0.031, -0.022] |
| **Age** |  |
| 18-35 years | Ref |
| 36-55 years | -0.007 [-0.009, -0.005] |
| 56+ years | -0.045 [-0.047, -0.043] |
| **Urbanicity** |  |
| Rural | Ref |
| Non-rural | 0.004 [0.001, 0.008] |
| Missing |  |
| **Response time** |  |
| 1^st^ quartile (0-4 mins) | Ref |
| 2^nd^ quartile (4-6 mins) | -0.016 [-0.018, -0.014] |
| 3^rd^ quartile (6-8 mins) | -0.029 [-0.031, -0.026] |
| 4^th^ quartile (8-18 mins) | -0.043 [-0.045, -0.041] |
| **First naloxone dose** |  |
| No administration | Ref |
| Lay administration only | 0.030 [0.025, 0.036] |
| Less than 2mg | -0.056 [-0.058, -0.054] |
| >= 2mg, <4mg | -0.020 [-0.022, -0.018] |
| 4mg or more | -0.010 [-0.013, -0.007] |
| **Level of care** |  |
| BLS | Ref |
| ALS | -0.001 [-0.003, 0.002] |
| **Location** |  |
| Private residence | Ref |
| Street/road | -0.028 [-0.031, -0.026] |
| Commercial | -0.011 [-0.014, -0.009] |
| Other | -0.050 [-0.052, -0.048] |
| **Time of day** |  |
| Day | Ref |
| Night | 0.002 [0.001, 0.004] |
| **Day of week** |  |
| Weekday | Ref |
| Weekend | -0.001 [-0.002, 0.001] |
| **Initial acuity** |  |
| Lower | Ref |
| Emergent | -0.015 [-0.017, -0.013] |
| Critical | 0.000 [-0.003, 0.004] |
| **Final acuity** |  |
| Lower | Ref |
| Emergent | -0.044 [-0.047, -0.041] |
| Critical | -0.054 [-0.058, -0.050] |
| **Responsiveness** |  |
| Never unresponsive measurements | Ref |
| Both responsive and unresponsive measurements | 0.002 [0.000, 0.004] |
| Only unresponsive measurements | -0.007 [-0.010, -0.004] |
| **Payment** |  |
| Insurance | Ref |
| Medicaid | -0.005 [-0.009, -0.002] |
| Medicare | -0.005 [-0.012, -0.001] |
| Other | 0.054 [0.050, 0.058] |
| Self Pay | 0.009 [0.006, 0.012] |
| No insurance | 0.020 [0.018, 0.022] |

Note: Average marginal effects and associated confidence intervals obtained from a logistic regression using non-transports as the outcome variable and including all presented covariates (n=710,263). Results are based on a pooled regression of five datasets imputed using the mice package in R with five iterations. Month-year and state fixed effects omitted from the table.
